# A Ubiquitous and Low-Cost Solution for Movement Monitoring and Accident Detection Based on Sensor Fusion

**DOI:** 10.3390/s140508961

**Published:** 2014-05-21

**Authors:** Filipe Felisberto, Florentino Fdez.-Riverola, António Pereira

**Affiliations:** 1 FCT (Fundação para a Ciência e a Tecnologia), Foundation for Science and Technology, Lisbon 1249-074, Portugal; 2 Higher Technical School of Computer Engineering, University of Vigo, Polytechnic Building, Campus Universitario As Lagoas s/n, Ourense 32004, Spain; E-Mail: fmfelisberto@uvigo.es; 3 INOV INESC INNOVATION, Institute of New Technologies of Leiria, P-2411-901, Leiria, Portugal; 4 Computer Science and Communications Research Centre, School of Technology and Management, Polytechnic Institute of Leiria, P-2411-901, Leiria, Portugal; E-Mail: apereira@ipleiria.pt

**Keywords:** inertial sensors fusion, Wireless Sensor Networks, Wireless Body Area Networks, motion recognition, rehabilitation, profiling

## Abstract

The low average birth rate in developed countries and the increase in life expectancy have lead society to face for the first time an ageing situation. This situation associated with the World's economic crisis (which started in 2008) forces the need of equating better and more efficient ways of providing more quality of life for the elderly. In this context, the solution presented in this work proposes to tackle the problem of monitoring the elderly in a way that is not restrictive for the life of the monitored, avoiding the need for premature nursing home admissions. To this end, the system uses the fusion of sensory data provided by a network of wireless sensors placed on the periphery of the user. Our approach was also designed with a low-cost deployment in mind, so that the target group may be as wide as possible. Regarding the detection of long-term problems, the tests conducted showed that the precision of the system in identifying and discerning body postures and body movements allows for a valid monitorization and rehabilitation of the user. Moreover, concerning the detection of accidents, while the proposed solution presented a near 100% precision at detecting normal falls, the detection of more complex falls (*i.e.*, hampered falls) will require further study.

## 1. Introduction

As of 2013, studies show that the reduction of the birth rate in developed countries associated with the increase of the life expectancy in both developed and in developing countries is causing the ageing of the World's population. For the first time in history, the World's population over 65 years old is larger than the population under 5 [1] and it is expected that by 2060 the population over 65 will have grown from the current 17.1% to 30.0% [2]. This situation entails two direct results, the first being an increase of health care necessities as, on average, with aging also comes a deterioration in health conditions; the second, a reduction of the workforce, which means there are more persons in need of support (both in terms of health care support and in terms of economic support) but less people available to provide that same support. By 2050 it is expected that the economic old-age dependency ratio in the European Union will have risen from 37% in 2003 to 70% [3].

At the same time, with the increased economic struggles faced by Western countries, many social problems have surfaced. The recession that started in late 2007 lead to a decrease in the gross domestic product (GDP) which was not accompanied by a similar decrease in health care expenditures (both public and private) [4].

In this context, the Elder Care platform was previously envisioned [5] with the goal of providing the necessary tools to mitigate the problems concerned with the need for personal monitoring of the elderly, both during activities of the daily living (ADL) as during rehabilitation processes. By delivering a system that is capable of constantly monitoring and analyzing the user's health status, the solution enables the elderly to continue living in their own homes instead of having to be institutionalized in a nursing home.

In this article, we tackle the specific problem of monitoring the user's movement. The field of human movement monitoring is widely studied and different types of solutions have been presented over the years. These solutions vary widely in terms of what is being monitored and how the monitoring is done. The most precise solutions rely on external monitoring systems composed of advanced image processing equipment to identify the users' movements, postures and infer the users' ADL [6,7]. However, there are still some limitations for these types of systems. The first, to be able to guarantee the correct detection it is necessary to make sure that there are no blind spots and that every room in the elderly person's home is equipped with the number of cameras required for the type of monitoring, so the high number of cameras necessary can become an economic detriment. Moreover, the monitoring ends if the user leaves his or her house. The second being the reluctance to accept a somehow Orwellian solution as an image processing solution requires waiving some of the user's privacy. For historic reference, one of the first projects for a fall detection system, was forced to shift from image processing to body placed sensors due to privacy concerns [8].

On the other hand, while an inertial sensor based solution is unable to have the same precision as computer vision in terms of motion capturing, it was already shown that by resorting to the correct sensor fusing techniques it is still a viable solution for movement recognition, and as it is placed on the user's body it doesn't suffer from spatial constraints and the user's sense of privacy is maintained [9]. Like with computer vision-based solutions, the ones based on inertial sensors also vary in degrees of complexity. In one side of the spectrum are the solutions whose sole function is to detect sporadic events and require a very small number of nodes and sensors, e.g., fall detection can be done with a single node [10]. In the middle of the spectrum are the solutions that track body actions and body postures using one [11] or two nodes [12,13], depending on the complexity of the algorithm chosen. Finally, in the end of the spectrum are the solutions, which not only track changes but are also able to discern differences between actions of the same type. The number of nodes necessary varies with the number of body parts being monitored, some implementations are specific to a single body part and require less sensors [14] while others require the full body to be monitored and need a large number of nodes [15]. In this part of the spectrum are the great majority of the applications concerning elite sport monitoring. These applications have a very low error tolerance, thus requiring both a high sampling rate (some as high as 500 Hz [16]) and the data to be externally processed. A good review of some of these applications can be found in [17].

This study intends to prove that a solution using inertial sensors supported by a wireless sensor network (WSN) can be scaled to a system that is viable for day-to-day use and still have a high precision in detecting deterioration in movement, help in rehabilitation and detect accidents. With this purpose, the rest of the paper is structured as follows: Section 2 provides a brief introduction to the WSN architecture used in this work. Section 3 presents the available sensor data and shows how it was fused to obtain the necessary information for the proposed solution. Section 4 explains how the system copes with the individualized characteristics of each user. Section 5 describes the supported events, which will be subsequently recognized and categorized. Section 6 presents the experimental protocol and analyzes the results obtained from the different tests carried out. Finally, Section 7 summarizes the mains conclusions extracted from the work and identifies future research lines.

## 2. Architecture of the System

When designing a system that relies on information being processed in a WSN, it is important not only to take into account the processing power of the sensor nodes, but also to evaluate how this extra processing will affect the node's autonomy. The architecture in which this solution relies is the result of the continuous improvements made to the BodyMonitor architecture [18]. While it was initially envisioned for a Fall Detection System (FDS), this constraint had already been taken into consideration and it was concluded that it was necessary to divide the processing tasks and relay some of the processing to an external system. This requires a rigorous study in advance to define what is processed internally and what is relayed, as heavy communication between the WSN and the external system has an even greater impact to the WSN's autonomy.

In this specific case, to be able to balance the cost between WSN processing and WSN communication, the data sampled by the sensor nodes are processed by the node itself but time series analyzes, both long and short term ones, are done on an external system. While the base principals from the initial architecture were kept, the architecture itself had to be redesigned taking into account the extra complexity of the new system. In the FDS each node was independent, relied solely on its own data and it did not require the direction component or any advanced filtering as it only used one sensor. In the present work there are two more sensors involved, there is more information that needs to be compared and the sensor nodes have to communicate between each other in order to access the state of the entire body. In addition, it is not possible to have a type of “one fits all” solution, as what can be normal values for a user can mean deterioration or improvement to another.

For intra-WSN communication the standard that most closely conforms to this project requisites is the IEEE 802.15.6 Body Area Network (BAN), which as of March 2014 is still under being drafted [19]. As the name infers, the standard was specially designed for communication between devices placed on the human body, and one of the most important aspects of this standard is its requirement for ultra-low power consumption [20]. As during the development of the node's prototype there was no available radio that already implemented the task group's 2011 draft [21], it was necessary to choose a testing alternative. The standard that was chosen for this purpose was the IEEE 802.15.4 Low Rate Wireless Personal Area Networks (LRWPAN) [22], which already has multiple implementations both closed and open, and a great variety of readily available radios. While there are clear differences in terms of energy autonomy between both standards, the ones attained by the LRWPAN were considered acceptable for a testing environment. At the same time, the constraints defined in the BAN draft were also taken into account in order to guarantee a future transition.

In terms of communication error handling and packet routing, both standards have already defined methods. As one of the objectives of this work is to minimize the main microcontroller utilization, it was given priority to techniques that were implementable in the radio itself.

**Figure 1.**
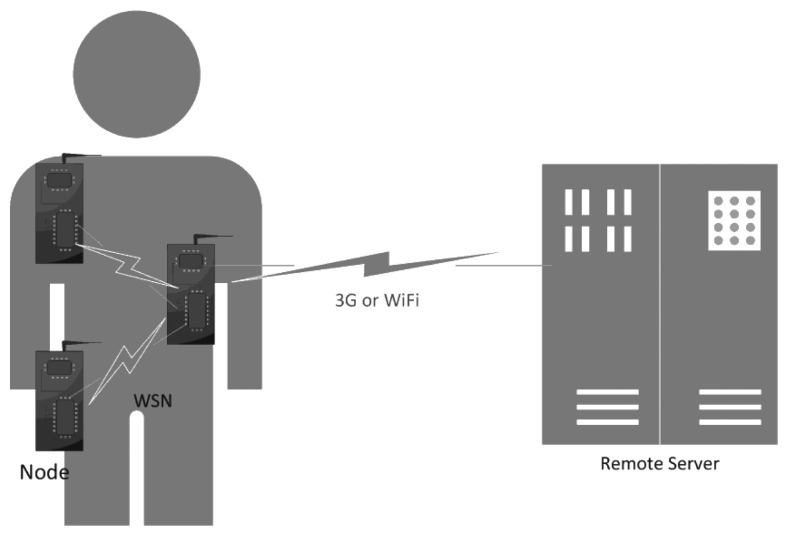
BodyMonitor architecture.

Thus, in summary, the proposed architecture keeps the bases from the previous architecture but its complexity is increased as new layers of processing and new capabilities are introduced, which require the use of improved information fusion technics [23]. In this context, Figure 1 shows a diagram of the BodyMonitor architecture followed by the description of its main components.

**Figure 2.**
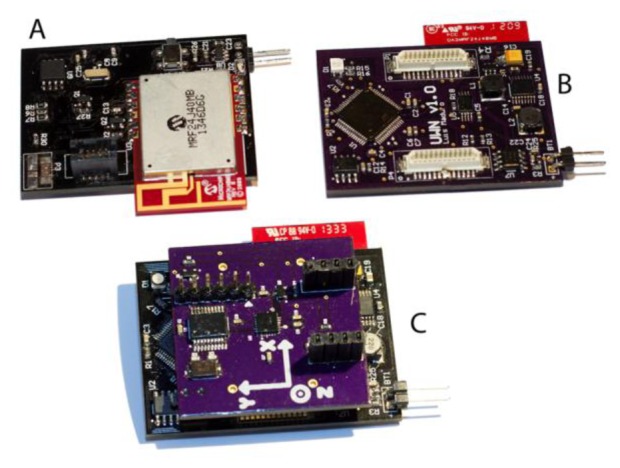
New node developed for the proposed solution. (**A**) radio side of the node containing the 802.15.4 compliant radio. (**B**) microcontroller and expansion port side. (**C**) node with the sensorial board attached.

- *Sensor node*: The sensor node (see Figure 2) is responsible for the acquisition and processing of information relative to the respective body area. It is the first layer of decision regarding the importance of the gathered information. The microcontroller base board measures 4.8 cm × 3.5 cm × 0.8 cm and weights 11 g, the expansion board measures 3.6 cm × 3.5 cm × 0.4 cm and weighs 5 g.
- *WSN*: In case of an event whose relevance cannot be decided solely using the information gathered by a single node, that node may request information from the remaining nodes and make a decision accordingly.
- *Remote server*: If even after contacting the remaining sensor nodes the data remains inconclusive, last samples from all sensors are sent to the remote server to be analyzed. The remote server is the one responsible to individualize each user's information, keep this information updated and to detect long-term changes.

## 3. Sensorial Data: Issues, Placement and Fusion

For data sources, the proposed solution relies on three sensor nodes, each one equipped with three tri-axial Microelectromechanical Systems (MEMS) sensors. The used sensors are an accelerometer, a gyroscope and a magnetometer. The selected gyroscope was the STMicroelectronics L3GD20, the magnetometer and the accelerometer functions were both provided by a STMicroelectronics LSM303DLHC unit.

As mentioned, by relying on sensors placed on the user's body the study is prone to errors natural to internal monitoring systems. In order to minimize inaccuracy, it is important to understand what the limitations of each sensor are and how to minimize the deviation it introduces.

During this and the following sections, and while describing movement and orientation, the global frame of reference has the *X* axis going backward to forward, the *Y* axis is left to right and the *Z* axis goes from bottom to top.

### 3.1. Accelerometer

The most commonly used sensor for both movement and orientation analysis is the accelerometer. In the past years, this type of sensor has become very precise and capable of handling a wide range of values. The selected accelerometer measures acceleration in all three axis. For the proposed application, the limitation of the accelerometer is that it measures proper acceleration, which is the acceleration relative to a resting referential [24], instead of directly measuring the movement's acceleration, making it unable by itself to differentiate the dynamic acceleration from gravity (*a⃗_prop_*=*a⃗_dyn_*+*g⃗*).

While integrating to obtain velocity, and in order to correctly understand the direction of the movement, gravity needs to be constantly subtracted from the previous vector. In order to accomplish this, data must be constantly updated from an initial reference. During slow changes of orientation the data loss from noise are minimal, but during fast activities, sudden changes of orientation or free fall it becomes impossible to correctly identify the node's orientation.

On the other hand, if the sum of all forces to which the system is being exposed are equal to the gravity force then the system is, most likely, in a stationary state. In this case, the formula used is *a⃗_prop_*=0⃗+*g⃗*; meaning gravity is the only acceleration vector to which the node is being subjected and, as gravity is a static vector, it can be used as a reference to calculate the orientation of the sensor node (although only pitch and roll can be obtained). These reasons make the accelerometer very precise for long-term analyses but prone to error on smaller erratic events.

### 3.2. Gyroscope

The gyroscope measures the angular velocity *ω⃗*. Unlike the accelerometer, its measurements are more absolute as there is no external information to be taken into account. Therefore, each independent sample is more precise than the one's obtained from the accelerometer. The first problem with the gyroscope is that the data it returns only represent the rate of change of the angular position, so to be able to know the sensor current angle it is necessary to have an initial reference. This initial orientation has to be continuously updated through integration of the gyroscope values. This leads to the second problem with gyroscopes, for a truly precise solution, it would be necessary to have an infinite number of samples, as for each sample not collected there is an increase in the aggregated error, this is known as angle random walk [25]. Moreover, as in all MEMS there is an inherent error. In the case of the gyroscopes the bias error causes an angular velocity to be detected even when there is no movement at all, which adds to the drift problem. Whereas while using the accelerometer it is possible to precisely infer the node's orientation from the gravity vector, the same cannot be done using the gyroscope, as there is no reference to use. As the gyroscope is unable to reset to an initial state it is not possible to discard all the accumulated errors, resulting in it being less precise on the long-term.

### 3.3. Magnetometer

Finally, the magnetometer measures the Earth's magnetic field in all three axis, allowing an accurate calculation of the sensor's orientation. In perfect conditions (*i.e.*, sensor perfectly calibrated to the geographic magnetic declination and in an area free of magnetic interference), with the magnetometer it is possible to have an instantaneous precision as high as the gyroscope with the long-term precision of the acceleration. Unfortunately, of all the considered sensors, the magnetometer is the one most prone to external interference. As the magnetometer measures all magnetic fields (not only the Earth's one) it is very sensitive to hard iron distortions, which are commonly created by the magnetic fields generated by other devices.

### 3.4. Sensor Placement

When selecting how many sensor nodes to use and where to place them, it was taken into account the ubiquity of the system, the costs to both deploy and maintain the WSN, and the easiness of use. A solution with a high number of nodes allows for a more complete monitoring, but it also implies a high deployment cost and greatly reduces its usability, jeopardizing its acceptance. Taking into consideration the economic disadvantages and the big percentage of digital exclusion, or even technophobia faced by this solution's target population [26] it was decided to use as few nodes as possible, without endangering its capability to detect abnormalities. This meant not monitoring body areas that while helpful in identifying user movement patterns, were not strictly required in the scope of this study (e.g., arms). Taking into consideration previous covered ideas, sensor nodes were finally restricted to the upper torso, hip and leg.

The upper torso sensor node (*Nt*) is primarily responsible for identifying bad back postures and recognizing when the user is lying. High accelerations in this area are always motive for further analysis, not only for being uncommon but also due to this node's proximity to the head.

The node placed in the hip (*Nh*) is the coordinator node, being responsible for maintaining the communication inside the WSN and to connect the WSN with the remote server. For its placement in a very stable position (under normal physical proportions in the center of mass [27]) this node is the sole responsible for keeping track of the body's movement as a whole.

The third node is located on one of the user's legs (*Nl*). If the user has a homogenous gait, the sensor is simply placed parallel to *Nh*, otherwise (e.g., due to a limp) the node is placed on the affected leg. While in [28] it was proven that the size of each step can be measured by accelerometer data being recorded from the hip, this solution allows for more precise monitoring of the way the gait is being performed, and is also invaluable in terms of identifying the leg position when resting.

In the following section, it will be discussed how it is possible to correctly identify movement patterns and variations by analyzing the data from the separate nodes and fusing the information among them.

### 3.5. Sensor Fusion

**Figure 3.**
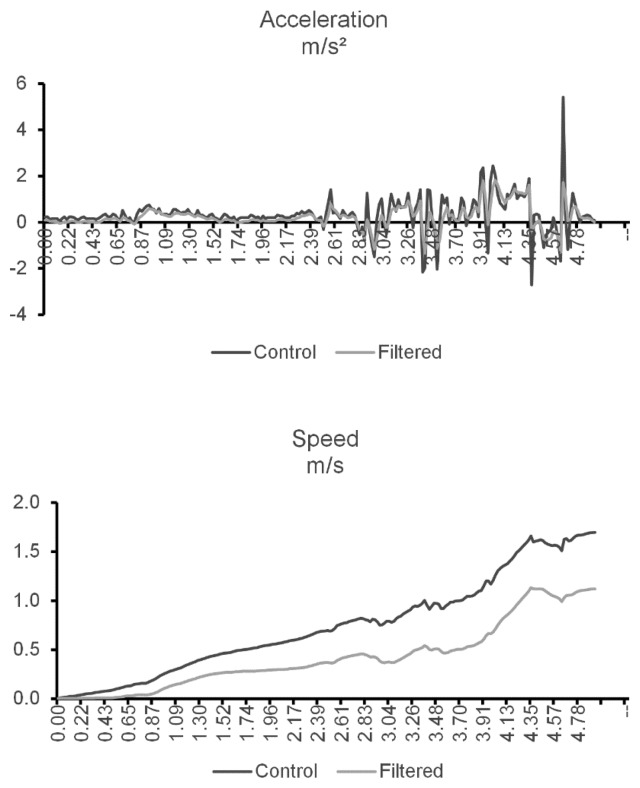
Acceleration and speed data with and without filtering.

While the proposed system does not require the high precision necessary for motion capture, it is still important to have a degree of confidence on the data being analyzed. For that reason, the data collected has to be filtered and normalized prior to be used. In a first step, the accelerometer data are refined using a median filter (with a window of five samples) in order to smooth out the noise. If the noise is not taken into account, it will propagate while integrating for speed. Figure 3 shows both unfused (control) and filtered data belonging to a simple test of walking for two meters in a straight-line (the samples were collected from the node placed on the hip). Due to the error prone nature of the double integration needed to obtain the distance, it will not be used in this project. This trial was a straight-line test designed specially to minimize noise, so it can be used to illustrate the difference between using filtered and unfiltered acceleration. By integrating both filtered and control speeds over the recorded test time, we get two very different distance values of 3.68 m and 2.10 m, respectively. This represents an accumulated error of 84% when using unfiltered acceleration and 5% when using filtered acceleration.

**Figure 4.**
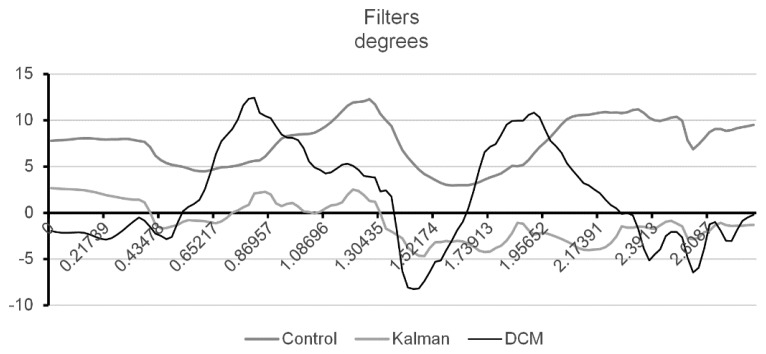
Study of the three different algorithms used for calculating orientation.

Afterwards, and in order to obtain a correct orientation of the node, data from the three sensors need to be fused. For the current project, three different fusing algorithms were studied: (*i*) Extended Kalman Filter (EKF), (*ii*) Direct Cosine Matrix (DCM) and (*iii*) a control algorithm which fused each individual sample ignoring past knowledge. Figure 4 represents the orientation obtained from fusing the sensorial data collected from the hip node whilst the user was walking with the least wobbling possible. The control algorithm showed a constant increase in error and the orientation continued to climb even after the spikes were recorded. Both DCM and EKF algorithms were able to handle the incremental error, but the EKF coped with noise spikes better, smoothing them out immediately. Both studies are straightforward explained in our previous work [9].

While fusing the three sensors, the ones that are mostly used are the accelerometer and the gyroscope. The magnetometer is used for data confirmation and normalization as its susceptibility to interferences from other devices makes it very prone to indoor errors, where the proposed solution will be mostly used.

Previous approaches that lack gyroscope and magnetometer use the absolute value of acceleration [10] permitting a direct subtraction of gravity by means of Equation (1):

(1) $$\left\|{\vec{a}}_{\text{dyn}}\right\|=\left\|\vec{a}\right\|-1=\sqrt{{a_{x}}^{2}+{a_{y}}^{2}+{a_{z}}^{2}}-1$$

However, by using this computation all the information about direction is lost. As the result of the pre-processing is a viable orientation quaternion ( qNG), it is possible to obtain the dynamic acceleration vector by removing the gravity component from the node's filtered acceleration. To be able to subtract the gravity vector, it is necessary to displace it from the global frame of reference (^*G*^*g⃗*) into the node's own frame (*^N^g⃗*). To do that, the rotation quaternion must be applied to gravity, but as a quaternion rotation can only be directly applied to another quaternion, it is first necessary to convert the vector into a pure imaginary quaternion. A pure quaternion is a quaternion with a zero scalar part, so the new quaternion is defined as *^G^g*=[0 0 0 1] and it can be rotated using Equation (2). Equation (2) is applicable because the rotation quaternion is a unit quaternion [29]:

(2) gN=qNGgGqNG*

The resulting quaternion will also have the scalar part zero, so its representation in *R*^3^ is the gravity vector *^N^g⃗*. The dynamic acceleration can now be calculated using Equation (3):

(3) $${\vec{a}}_{\text{dyn}}={\vec{a}}_{\text{prop}}-\vec{g}$$

By integrating the dynamic acceleration vector over time (using Equation (4)), we obtain the movement's velocity. Unfortunately, even after filtering the data it still presents a degree of error, so in the long-term, velocity becomes unusable and it is best suited to support accident detection:

(4) $$\vec{v}(t)=\int {\vec{a}}_{\text{dyn}}(t)dt$$

The sensor node's orientation and its dynamic acceleration vector make up for the necessary information for studying the movement of the corresponding body part.

### 3.6. Internal and External Sensor Fusion

The wireless nodes collect information with reference to themselves and not the body part to which they are attached, so directly mixing information between users would lead to misclassification, as users with different body characteristics would have different orientation values for the same postures.

The external system is only required during an initial calibration process where each user's posture is calculated using Microsoft Kinect SDK. To avoid errors, data from both computer vision and wireless node are averaged during a period of ten seconds to define the initial orientation quaternions (Equation (5)) [30]:

(5) $$q_{cv},{\text{q}}_{wn}$$

During regular use, to obtain the normalized orientation it is first calculated the rotation quaternion that would rotate the wireless node from its initial state to the current one (*q*) (Equation (6)). This rotation is then applied to the computer vision quaternion (Equation (7)) and the resulting orientation can be compared with other user's normalized orientation:

(6) $$q_{\text{rot}}=q\times {q_{wn}}^{-1}$$

(7) $$q_{\text{norm}}=q_{cv}\times q_{\text{rot}}$$

A similar approach was used in [31] to fuse gyroscope data and computer vision markers in order to better monitor the movement of a tennis player's arm.

## 4. Profiles

To be able to accept any user (regardless of his size, weight or sex) the system uses a combination of two types of profiles: (*i*) an ideal profile also referred as *default profile*, which contains the ideal values for the user's age, height, weight and health condition, which are defined by the supervising physician accompanying the user and (*ii*) a *individual profile*, storing information unique to each user together with the user's deviations from the default profile.

### 4.1. Default Profile

The information stored in the default profile is shown in Table 1.

### 4.2. Individual Profile

Additionally to the deviation from the default profile (*Pu*_1_,…,*Pu*_7_), each user profile contains data unique to each user. Table 2 specifies this information.

### 4.3. Inferred Information

In the same way as *velocity* could be inferred by fusing the information from multiple sensors, additional information can be obtained by using the physical quantities already calculated in conjunction with the existing profile information. This extra information is presented in Table 3.

A step is calculated by measuring the time between peak-to-peak acceleration values from the hip node. The step length (*Slt*) is calculated integrating velocity over the time interval of the step. The step cadenced (*Scd*) is the number of detected steps per second. The step force (*Sf*) is calculated using the vertical acceleration from the leg node (this does not represent the actual force as some of it is absorbed by both the foot and lower leg, but can still be used to detect deterioration or improvements). For energy conservation reasons, both *Slt* and *Sf* are only processed for the first step of each sample.

## 5. Events

During constant monitoring of the user, the sensor node will trigger an *event* whenever it detects a change in the *state* of its movement or orientation, or when the data being analyzed are different from what is defined in the user's profile. There are three types of events used in this work: (*i*) state changes, (*ii*) anomalies and (*iii*) alerts.

**Figure 5.**
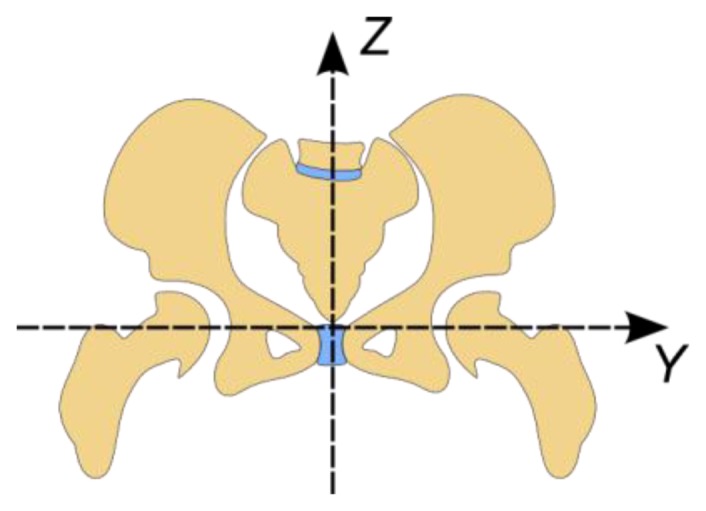
Reference system used for the human body.

In this section and while describing the human body's rotation, the frame of reference used has its origin in the hip joint area (Figure 5) and its axes follow the same rules defined for the global frame of reference.

Also, for rotation nomenclature we will use those terms normally associated with aviation. Accordingly, roll is a rotation over the *X* axis, pitch over the *Y* axis and yaw over the *Z* axis.

### 5.1. State Change

To be able to confirm and/or differentiate between events, it is necessary to know the user's state. This requires that each sensor node, as well as the WSN itself, keeps track of the information regarding the state of the movement of both the entire body and individual nodes (see Table 4) and the body posture (see Tables 5 and 6).

The algorithm used to differentiate between activity and inactivity is based on the Acceleration Moving Variance Detector function (Equation (8)) defined in [32], which is based on the work [13]. This algorithm compares the variance of a window of acceleration samples to a pre-defined threshold, and movement is detected if the variance surpasses this threshold. In our adaptation, the acceleration used (*a*) is the already filtered dynamic acceleration instead of raw acceleration. The window size (*N*) used is 20 samples and the threshold (*γ*) 0.0013:

(8) $$\frac{1}{N}\sum_{k=1}^{N}{\left\|a_{n}-{ā}_{k}\right\|}^{2}<\gamma$$

The full body movement state is defined by the hip node's last state.

While the full body movement state was dependent on solely the information from one node (hip), the orientation state is defined by the information from both the torso and leg nodes. Each node only informs the WSN once for each sampling period, only if the node's final orientation would trigger a full body state change. In Table 5 are defined the state transitions functions for each of the three possible states (Standing (*O_St_*), Lying (*O_L_*) and Seated (*O_Si_*)). Moreover, with the goal of simplifying the comprehension of Table 5, auxiliary Table 6 shows the sub equations used to define the torso and leg's orientation as horizontal or vertical.

### 5.2. Anomalies

An anomaly is a discrepancy between the data analyzed by a sensor node and what has been defined in the user's individual profile. Anomalies can be detected based solely in the data from one sensor or it may require data from the WSN. Up to now, the supported anomalies are those showed in Table 7.

Whenever a sensor node detects an anomaly it will always be reported back to the remote server, so that it can be further analyzed in order to verify the existence of an alert. Before sending the anomaly the node buffers it for a predefined threshold (10 s), so extra information can be gathered. Moreover, for anomalies 1 to 3 (see Table 7) if after 10 seconds no other event was fired and the condition that led to the anomaly was corrected, the anomaly is stored and it is only sent during scheduled information updates. This is necessary, as communication with the remote server is very burdensome to the batteries.

### 5.3. Alert

Alerts are in fact the specifications of the anomalies, which for motives of energy conservation and lack of processing power have to be identified after the information is analyzed in the remote server. Contrary to the anomaly, when discerning alerts it is taken into consideration samples that were collected prior and after the event (series analyses), so more than one anomaly may be necessary to trigger a given alert. The types of alerts already supported in our system are showed in Table 8.

The rule of not sending anomalies directly to the remote server if the problem has been corrected, leads to alerts *Al*_3_, *Al*_4_ and *Al*_3_ serving for posterior re-education of the way of correctly preceding rather than to actually correcting as it is taking place.

## 6. Experimental Results

To demonstrate the validity of our proposal, a set of different tests were designed and conveniently carried out. These tests were divided into two major groups: (*i*) the first group consists of experiments to verify if the states are being correctly identified while (*ii*) the second one consists of experiments to validate the abnormality and alert detection process.

### 6.1. General Experiment Procedure

**Figure 6.**
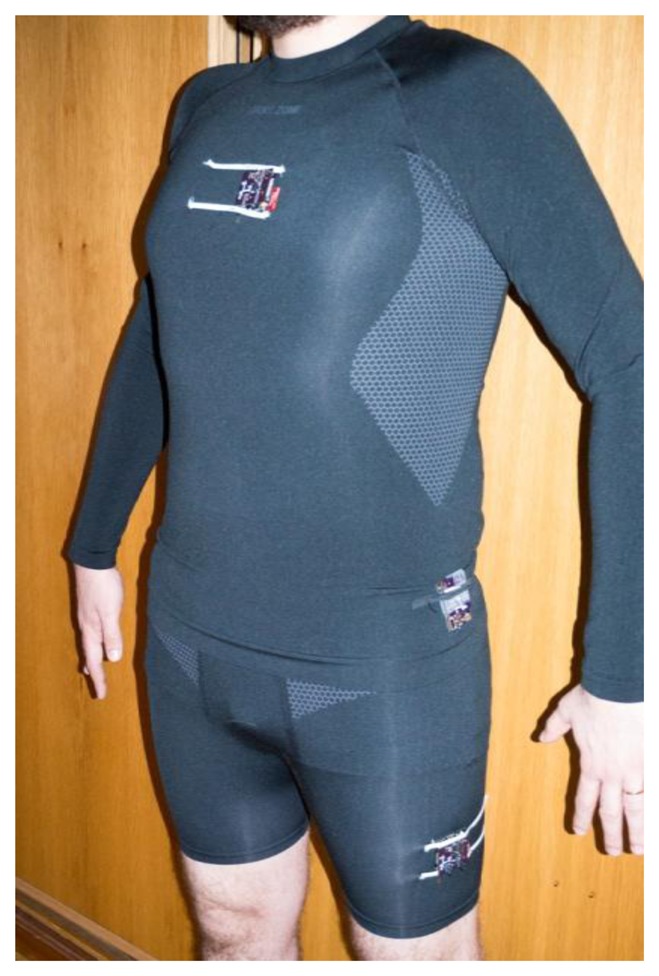
Volunteer using the recording platform.

As with any testing scenario that involves human interaction, the WSN data will have slight variations, even when the same person repeats the procedure. In this context, and with the goal of both having data to be reused and the experiments revalidated, all the experiments used pre-recorded data captured using the WSN. Moreover, to guarantee an adequate sampling rate data were captured with a sampling rate of 100 Hz, twice as high as what is considered the optimum for human movement recognition [11]. Each of the ten volunteers conducted every test twenty times. To guarantee a maximum number of valid tests, each group of tests was validated using the developed software. In case of recording errors (due to prototype malfunction or user fault) those tests were immediately repeated under the same conditions. In terms of sex distribution, five volunteers of each genre were selected. The average age of the volunteers was of 27.2 years with a standard deviation of 3.73. The average height and weight were respectively 1.62 meters and 72.6 kilograms with a standard deviation of 0.08 and 16.98, respectively. Figure 6 shows how each node (chest, hip and leg) was placed in a volunteer for sensorial data recording.

For practical reasons, the initial tests were run on an x86 hardware architecture instead of directly on the WSN. When the algorithms entered a stable state, they were ported to the WSN architecture and the experiments were again run, now using the actual nodes. For the current work all the experiments were done by healthy and young volunteers, as some of the rougher tests could cause injuries to a more elderly user.

### 6.2. Movement State Experiments

It is important for the system to be able to correctly detect any transition between states, otherwise it will not be able to correctly identify abnormalities. This is true because the system requires the knowledge of the current state to be able to identify the actual type of abnormality and, in some cases, to define the degree of importance of an alert (e.g., if the user's state after an impact is *laying*, it is given a higher importance to the alert than if the state is *standing*).

The movement states are *Stationary* and *Moving*. It is important to keep in mind that while one or more sensor nodes may detect movement changes, it may not mean the user is moving. For example, the user might be sitting down and still be shaking his leg nervously. In order to test if the system is able to discern if the user is moving or he is stationary different test scenarios (showed in Table 9) were created.

### 6.3. Orientation State Experiments

The objective of the tests presented in Table 10 is to verify (*i*) if the system is able to correctly recognize normal human positions, (*ii*) identify the transition between these positions and (*iii*) the precision at which each body parts' orientation is being calculated.

### 6.4. Alerts

The tests described in Table 11 were designed to verify if the system is able to detect alert in the user's states. Like in body orientation scenarios, it is not only important to detect that there is a problem, but also to know what the accuracy of the values being measured is.

### 6.5. Discussion

**Figure 7.**
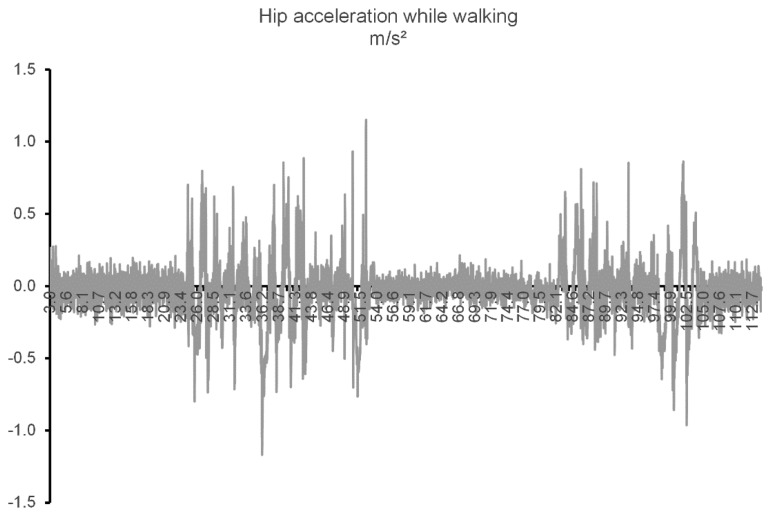
Forward acceleration. Movement sequence is: still, walking, still, walking and still.

The experiments have shown that the filters applied enabled the system to correctly detect the movement changes in all the test cases, being able to distinguish between individual node activity and full body movement. Nevertheless, it was also evident that there was a lag between the change in movement and the system actually detecting it. This is caused by the noise filtering toning down movement changes slightly. This fact did not affect the current tests, but if the necessity arises for a more precise detection of activity transitions, we are pondering whether to adding a node (or second microcontroller in an existing node) reserved for movement processing so that a more advanced algorithm may be used. Figure 7 depicts data from a WSW experiment showing how inconstant the acceleration readings are, even when the user is stationary.

**Figure 8.**
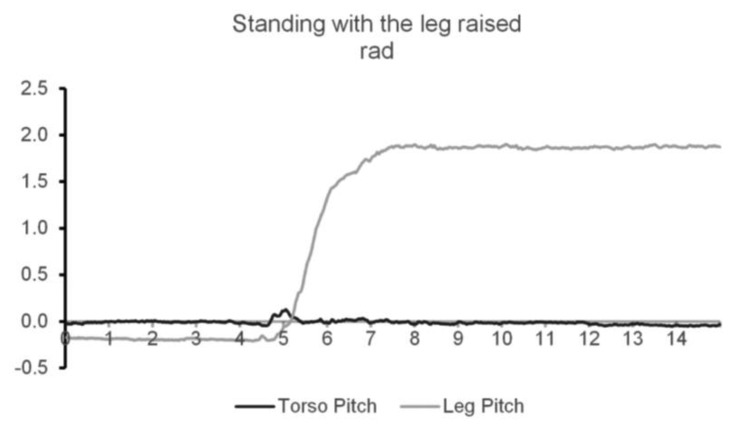
Processed pitches from the Leg and Torso nodes while standing with the leg raised.

**Figure 9.**
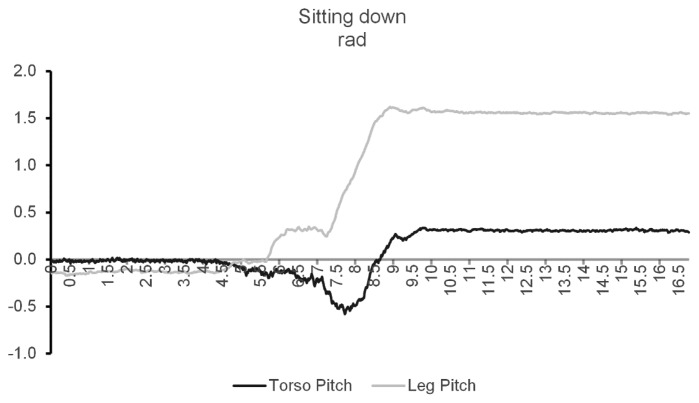
Processed pitches from the Leg and Torso nodes while sitting down.

In the orientation state experiments, the only cases that caused misclassifications were the ones specially designed to be ambiguous. The test that required the user to be standing with the sensor leg raised perpendicular to the torso caused the system to misclassify the orientation as sitting. This similarity can be confirmed by observing both Figures 8 and 9.

After pondering both the impact of such misclassification against the increase in cost of the immediate solution (*i.e.*, using a wireless node in each leg and require both nodes to confirm a horizontal state in order to classify the full body orientation as seated) and the fact that such ADLs are extremely rare in the target age demographics, it was decided that the minor impact to the system brought by this type of misclassification did not justify the increase in the system cost and energy consumption required by the extra communication.

In the experiments where the user was asked to slowly slide in the chair there were also misclassifications this time as laying down. It is important to notice that in between the changes of state the abnormalities for incorrect torso orientation were fired, so while there were misclassifications in terms of body orientation the system was still able to fulfill its objective of detecting incorrect postures.

**Figure 10.**
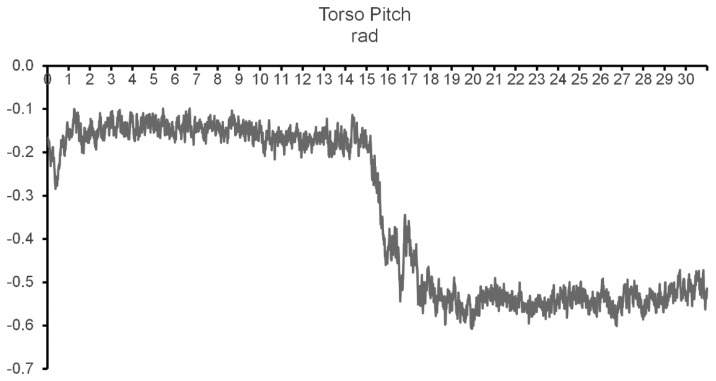
Processed pitch from the Torso node while walking normally and simulating a small hump.

In regard to the precision of incorrect activity detection tests, it varied between test's repetitions but was always higher than 70%. As this type of alert is to be used during long-term analyses a precision of 100% is not necessary, so the precision attained during the tests is enough to guarantee that the movement deterioration would be detected in an early stage. Figure 10 shows data comparing the torso's pitch, first while walking normally and then (after 16 s) with a forward rotation of the torso simulating a small hump.

This type of tolerance does not apply to fall detection, which requires a very high precision as what is being analyzed is the isolated event. In regard to the normal fall tests, the precision obtained was 99.5%. These tests were done not to validate the algorithm itself, as this was already done in previous works, but to warrant that the different hardware do not affect its precision.

On the other hand, the hampered fall tests revealed a low precision of approximately 59%. This was the highest viable precision, as if the sensibility of the algorithm was lowered, some of the normal ADLs (specially sitting down) started to trigger hampered falls alerts. None of the testers' attributes (age, genre or physical characteristics) were demonstrated as having any statistically meaningful impact on the misclassifications.

Finally, Table 12 shows the results for each individual experiment carried out. The average passing percentage of these tests was of 85.6% with a standard deviation of 0.3. However, if the outlier test (corresponding to the rare ADLs) is not taken into consideration, then the passing average increases to 94.3% with a standard deviation of 0.1.

## 7. Conclusions and Future Work

This study excels not only by surpassing the precision of the previously cited WSN projects in monitoring users' movements, but on providing the necessary tools to help with long-term rehabilitation and long-term problem identification. By restricting the focus of the system, it was also possible to reduce the number of wireless nodes, therefore minimizing the deployment cost of this solution compared with other WSN projects, and to make it almost as ubiquitous as the solutions that use image processing, without their high cost, privacy concerns and space restrictions.

Regarding the detection of accidents, this solution presents the same high precision as the more prominent studies of the area, but also suffers from the same limitations concerning the detection of hampered falls. In this aspect, one of the few projects with a good performance uses a hybrid solution, requiring both MEMS sensors and computer vision tag and with that also having the limitations inherent to both systems [35].

Additional techniques and sensor types are being evaluated to further advance on the field of remote monitoring, without compromising the system's usability or substantially increasing the deployment costs. The new techniques include the use of custom Beliefs-Desires-Intentions (BDI) agents. A multi-agent system will be designed using the JADE [36] agent framework and we will use both deliberative agents and CBR techniques in order to improve the agents learning capabilities [37]. With them, it is expected to automatically identify new variables and enable even more individualized profiles and rules, making the solution capable of adapting to each user's special necessities. By relying on a JADE-based multi-agent system, it will be possible to migrate from the remote server to a distributed system paradigm, allowing the execution of agents in different machines spread over different networks.

In regards to the sensors being equated for generic health monitoring, the one which stands out for the benefits it brings to movement monitoring (especially accident detection) is the heart rate monitor. With both the multi-agent system and the new health monitoring sensors it is expected to be able to discern any normal ADLs from accidents and obtain high precision scores in hampered fall detection.

## Figures and Tables

**Table 1.** Variables present in the default profile.

**Id**	**Description**
*P*_1_	Ideal orientation of the torso while standing (*P*_1,*x*_,*P*_1,y_)
*P*_2_	Ideal orientation of the torso while walking (*P*_2,*x*_,*P*_2,y_)
*P*_3_	Ideal orientation of the torso while sitting (*P*_3,*x*_,*P*_3,y_)
*P*_4_	Range of speed values while walking ( ](*P*_4,min_,*P*_4,max_)[ )
*P*_5_	Range of step cadence while walking ( ](*P*_5,min_,*P*_5,max_)[ )
*P*_6_	Step length (ratio)
*P*_7_	Step force (ratio)

**Table 2.** Variables specific to each user.

**Id**	**Description**
*Pu*_8_	Weight
*Pu*_9_	Height
*Pu*_10_	Leg length

**Table 3.** Calculated variables.

**Id**	**Description**
*Slt*	Step length
*Scd*	Step cadence
*Sf*	Step force

**Table 4.** Movement states.

**State**	**Description**
*M_M_*	Movement state–Moving
*M_S_*	Movement state–Stationary

**Table 5.** Orientation states and respective state change equations.

**State**	**Description**	**Transition State**	**Equation**
*O_St_*	Orientation state–Standing	*O_L_*	*T_h_* ∧ *L_h_*
		*O_Si_*	*L_h_*

*O_L_*	Orientation state–Lying	*O_St_*	*T_v_* ∧ *L_v_*
		*O_Si_*	*T_v_*

*O_Si_*	Orientation state–Seated	*O_St_*	*L_v_*
		*O_L_*	*T_h_*

**Table 6.** Body part orientation equations.

**Orientation**	**Description**	**Equation**
*T_h_*	Horizontal Torso	$\forall Nt\in \{Nt_{\text{roll}},Nt_{\text{pitch}}\}:\frac{2\pi }{3}\leq \left\|Nt\right\|\geq \frac{\pi }{3}$
*T_v_*	Vertical Torso	$\forall Nt\in \{Nt_{\text{roll}},Nt_{\text{pitch}}\}:\frac{\pi }{4}\leq \left\|Nt\right\|$
*L_h_*	Horizontal Leg	$-\frac{\pi }{3}\geq Nl_{\text{roll}}\geq \frac{\pi }{6}\wedge \left\|Nl_{\text{pitch}}\right\|\geq \frac{\pi }{6}$
*L_v_*	Vertical Leg	$-\frac{\pi }{6}\leq Nl_{\text{roll}}\leq \frac{\pi }{12}\wedge \left\|Nl_{\text{pitch}}\right\|\leq \frac{\pi }{12}$

**Table 7.** Description and equations of the events characterized as anomalies.

**Id**	**Description**	**Equation**
*An*_1_	Torso's inclination	*Nh_roll_* > *P*_1,x_ ∨ *Nh_pitch_* > *P_1,y_* → *An*_1_
*An*_2_	Incorrect gait	*Slt* > *Pu*_6_.*Pu*_9_ ∨ *Pu*_9,min_ < *Scd* > *Pu*_9,max_ → *An*_2_
*An*_3_	Contact force of the step	$Nl_{G}^{N}{\vec{a}}_{\text{dyn},z}>Sf\to An_{3}$
*An*_4_	Excessive vertical acceleration	N∈{Nh,Nt,Nl},a→GNdyn,z<0∧‖a→GNdyn‖>1g→An4
*An*_5_	Excessive body velocity with a descendent direction	*^G^v⃗_dyn,z_* < 0 ∧ ‖*^G^v⃗_dyn,x_*‖ <‖ *^G^v⃗_dyn,z_*‖ >‖*^G^v⃗_dyn,x_*‖ ∧‖*^G^a⃗_dyn_* ‖>1g → *An*_5_

**Table 8.** Description and triggering effects of the events characterized as alerts.

**Id**	**Description**	**Trigger**
*Al*_1_	Normal fall	Triggered by *An*_4_ and *An*_5_. To guarantee a high certainty both Bourke's approach [33], which relies on the trunk's vertical velocity, and the previously referred solution is used, which applies an acceleration threshold in the WSN and logistic regression in the remote server to confirm the actual fall [34].
*Al*_2_	Hampered fall	Triggered by *An*_4_, *An*_5_ and requires *M_s_*. Being the current state *M_s_* and *x* the number of occurrences of *An*_4_∨ *An*_5_ in the last 3 seconds, *x* > 3 → *Al*_2_.
*Al*_3_	Incorrect posture while seated	Triggered by *An*_1_ and requires *O_st_*. After three consecutive *An*_1_ without a change of orientation → *Al*_3_. After the identification of *Al*_3_, the WSN is notified to stop sending anomalies of the type *An*_1_ until there is a change of posture.
*Al*_4_	Gait deterioration	Triggered by *An*_2_, *An*_3_ and requires *M_M_*. The procedure is the same as in *Al*_3_.

**Table 9.** Description of movement state experiments.

**Experiment**	**Description**	**State**
Walking	The test subject walks in a natural in an L shaped route back and forth for one minute.	*M_M_*
Still	The test subject remains stationary standing up, sitting down and laying down for one minute.	*M_S_*
Walking–Still–Walking (WSW)	The test subject does the same route, but this time before turning around in the end of the route, he should remain still for 10 seconds. After the pause, he resumes walking, each complete route is done 5 times.	*M_M_, M_S_*
Standing not still (SNS)	While standing upright, the user is requested to make the following movements without walking: (i) Catch an object from the floor; (ii) Place that object in a high place; (iii) At a normal pace, flex the leg raising it from the ground; (iv) Rotate his upper body from one side to another.	*M_S_*

**Table 10.** Description of orientation state experiments.

**Experiment**	**Description**	**State**
Standing	(i) Control, standing in the user' normal position; (ii) While standing, rise the sensor leg.	*O_St_*
Seated	(i) Control, sitting with straight back in the chair. (ii) Slowly slide in a *chaise longue*, into an almost horizontal position.	*O_Si_*
Lying down	(i) Lying on the back; (ii) Lying with the chest down; (iii) Lying on the side, straight and curled.	*O_L_*

**Table 11.** Description of alert experiments.

**Experiment**	**Description**	**Alert**
Incorrect walking	(i) Using the walking scenario, the user is requested to walk the same L shaped route this time simulating a small hump. (ii) Same as above but this time changing the gait by using an incorrect step width. (iii) Again, this time using an excessive force with the right foot and on the way back from the L shaped route with the left foot.	*Al*_4_
Incorrect sitting	(i) Using the sitting scenario, but this time sitting while making a positive angle with the chair; (ii) Same as before, but this time making a negative angle with the chair.	*Al*_3_
Incorrect activity	The user is requested to pick up an object from the ground, first in a correct manner, by bending the knees and not the back, then in an incorrect manner.	*Al*_5_
Normal fall	The type of fall being tested is the one where the accident consists on the accident of falling directly to the ground without any type of intermediate deceleration.	*Al*_1_
Hampered fall	This type of fall is harder to simulate has the user has to hit an intermediate obstacle that reduces its fall, without harming itself and yet produce valid data.	*Al*_2_

**Table 12.** Summary of results for each individual test.

**Group**	**Test**	**Pass Percentage**
*Movement state*	Walking	100.00%
	Stopped	100.00%
	WSW	99.00%
	SNS (*i*)	100.00%
	SNS (*ii*)	100.00%
	SNS (*iii*)	98.50%
	SNS (*iv*)	99.00%

*Orientation state*	Standing (*i*)	100.00%
	Standing (*ii*)	6.50%
	Seated (*i*)	100.00%
	Seated (*ii*)	0.00%
	Lying down (*i*)	100.00%
	Lying down (*ii*)	100.00%

*Alert*	Incorrect walking (*i*)	100.00%
	Incorrect walking (*ii*)	81.50%
	Incorrect walking (*iii*)	74.00%
	Incorrect sitting (*i*)	95.50%
	Incorrect sitting (*ii*)	97.50%
	Incorrect activity	88.50%
	Normal fall	99.50%
	Hampered fall	59.00%

## References

[1] Kinsella K., He W. (2009). An Aging World: 2008 International Population Reports.

[2] Giannakouris K. (2010). Ageing characterises the demographic perspectives of the European societies. Statistics.

[3] Carone G., de las Comunidades Europeas (2005). Dirección General de Asuntos Económicos y Financieros, C. Long-term Labour Force Projections for the 25 EU Member States: A Set of Data for Assessing the Economic Impact of Ageing.

[4] Keegan C., Thomas S., Normand C., Portela C. (2013). Measuring recession severity and its impact on healthcare expenditure. Int. J. Health Care Finance Econ..

[5] Marcelino I., Barroso J., Bulas Cruz J., Pereira A. Elder Care Architecture.

[6] Poppe R. (2010). A survey on vision-based human action recognition. Image Vis. Comput..

[7] Weinland D., Ronfard R., Boyer E. (2011). A survey of vision-based methods for action representation, segmentation and recognition. Comput. Vis. Image Underst..

[8] Lord C.J., Colvin D.P. (1991). Falls in the Elderly: Detection and Assessment. Proc. Annu. Int. Conf. IEEE Eng. Med. Biol. Soc..

[9] Felisberto F., Costa N., Fdez-Riverola F., Pereira A. (2012). Unobstructive Body Area Networks (BAN) for efficient movement monitoring. Sensors.

[10] Bourke A.K., O'Brien J.V, Lyons G.M. (2007). Evaluation of a threshold-based tri-axial accelerometer fall detection algorithm. Gait Posture.

[11] Mathie M.J., Lovell N.H., Coster A.C.F., Celler B.G. Determining activity using a triaxial accelerometer.

[12] Culhane K.M., Lyons G.M., Hilton D., Grace P.A., Lyons D. (2004). Long-term mobility monitoring of older adults using accelerometers in a clinical environment. Clin. Rehabil..

[13] Veltink P.H., Bussmann H.B.J., de Vries W., Martens W.L.J. (1996). Detection of static and dynamic activities using uniaxial accelerometers. IEEE Trans. Rehabil. Eng..

[14] Zhou H., Stone T., Hu H., Harris N. (2008). Use of multiple wearable inertial sensors in upper limb motion tracking. Med. Eng. Phys..

[15] González-Villanueva L., Alvarez-Alvarez A., Ascari L., Trivino G. (2013). A tool for linguistic assessment of rehabilitation exercises. Appl. Soft Comput..

[16] Dadashi F., Crettenand F., Millet G.P., Aminian K. (2012). Front-crawl instantaneous velocity estimation using a wearable inertial measurement unit. Sensors.

[17] James D., Busch A., Ohgi Y. (2009). Quantitative assessment of physical activity using inertial sensors. Digit. Sport Perform. Enhanc. Compet. Evol..

[18] Felisberto F., Moreira N., Marcelino I., Fdez-Riverola F., Pereira A. Elder Care's Fall Detection System.

[19] Kwak K.S., Ullah S., Ullah N. An overview of IEEE 802.15.6 standard.

[20] Reichman A. Standardization of body area networks.

[21] IEEE 802.15 WPAN Task Group 6 (TG6) Body Area Networks. http://www.ieee802.org/15/pub/TG6.html.

[22] IEEE 802.15 WPAN Task Group 4 (TG4). http://www.ieee802.org/15/pub/TG4.html.

[23] Felisberto F., Maduro L., Fdez-Riverola F., Barroso J., Pereira A. Information fusion for body area networks in healthcare environments.

[24] Taylor E.F., Wheeler J.A. (1992). Spacetime Physics.

[25] Stockwell W. Angle Random Walk. http://www.xbow.com/Literature/Application_Notes_Papers/Angle_Rom_Walk_Estimation_for_Rate_Gyros.pdf.

[26] FreshMinds (2008). Understanding Digital Exclusion.

[27] Lippert L. (2011). Clinical Kinesiology and Anatomy.

[28] Lee J.B., Mellifont R.B., Burkett B.J. (2010). The use of a single inertial sensor to identify stride, step, and stance durations of running gait. J. Sci. Med. Sport.

[29] Kuipers J.B. (1999). Quaternions and Rotation Sequences: A Primer with Applications to Orbits, Aerospace and Virtual Reality.

[30] Markley F., Cheng Y., Crassidis J., Oshman Y. (2007). Quaternion averaging. J. Guid. Control. Dyn..

[31] Ahmadi A., Rowlands D.D., James D.A. (2010). Development of inertial and novel marker-based techniques and analysis for upper arm rotational velocity measurements in tennis. Sport. Eng..

[32] Olivares A., Ramírez J., Górriz J.M., Olivares G., Damas M. (2012). Detection of (in)activity periods in human body motion using inertial sensors: a comparative study. Sensors.

[33] Bourke A.K., O'Donovan K.J., Nelson J., OLaighin G.M. (2008). Fall-detection through vertical velocity thresholding using a tri-axial accelerometer characterized using an optical motion-capture system. Conf. Proc. IEEE Eng. Med. Biol. Soc..

[34] Felisberto F., Felgueiras M., Seco A., Fdez-Riverola F., Pereira A. (2011). Application of Statistical Methods to Improve an Acceleration Based Algorithm. CENTERIS 2011, Part III, CCIS 221 proceedings.

[35] Dovgan E., Luštrek M., Pogorelc B., Gradišek A., Burger H., Gams M. (2011). Intelligent elderly-care prototype for fall and disease detection. Slovenian Med. J..

[36] Java Agent Development Framework. http://jade.tilab.com/.

[37] Laza R., Pavón R., Corchado J.M. A reasoning model for CBR_BDI agents using an adaptable fuzzy inference system.

